# The Association of Prenatal Diagnoses with Mortality and Long-Term Morbidity in Children with Specific Isolated Congenital Anomalies: A European Register-Based Cohort Study

**DOI:** 10.1007/s10995-024-03911-9

**Published:** 2024-03-04

**Authors:** Anna Heino, Joan K. Morris, Ester Garne, Silvia Baldacci, Ingeborg Barisic, Clara Cavero-Carbonell, Laura García-Villodre, Joanne Given, Sue Jordan, Maria Loane, L. Renée Lutke, Amanda J. Neville, Michele Santoro, Ieuan Scanlon, Joachim Tan, Hermien E. K. de Walle, Sonja Kiuru-Kuhlefelt, Mika Gissler

**Affiliations:** 1 Department of Knowledge Brokers, Finnish Institute for Health and Welfare, Mannerheimintie 166, 00270 Helsinki, Finland; 2 Department of Pediatrics and Adolescent Medicine, Lillebaelt Hospital, University Hospital of Southern Denmark, Kolding, Denmark; 3 Population Health Research Institute, St George’s, University of London, London, UK; 4 Rare Diseases Research Unit, Foundation for the Promotion of Health and Biomedical Research in the Valencian Region, Valencia, Spain; 5 Faculty Health and Life Sciences, Swansea, Wales; 6 Centre of Excellence for Reproductive and Regenerative Medicine, Children’s Hospital Zagreb, Medical School University of Zagreb, Klaiceva 16, 10000 Zagreb, Croatia; 7 Department of Genetics, University of Groningen, University Medical Center Groningen, Groningen, The Netherlands; 8 Institute of Nursing and Health Research, Ulster University, Coleraine, UK; 9 IMER Registry (Emilia Romagna Registry of Birth Defects), Center for Clinical and Epidemiological Research, University of Ferrara, 44121 Ferrara, Italy; 10 Unit of Epidemiology of Rare Diseases and Congenital Anomalies, Institute of Clinical Physiology, National Research Council, Pisa, Italy

**Keywords:** Congenital diaphragmatic hernia, Gastroschisis, Prenatal diagnosis, Spina bifida, Transposition of great arteries

## Abstract

**Objectives:**

To compare 5-year survival rate and morbidity in children with spina bifida, transposition of great arteries (TGA), congenital diaphragmatic hernia (CDH) or gastroschisis diagnosed prenatally with those diagnosed postnatally.

**Methods:**

Population-based registers’ data were linked to hospital and mortality databases.

**Results:**

Children whose anomaly was diagnosed prenatally (n = 1088) had a lower mean gestational age than those diagnosed postnatally (n = 1698) ranging from 8 days for CDH to 4 days for TGA. Children with CDH had the highest infant mortality rate with a significant difference (p < 0.001) between those prenatally (359/1,000 births) and postnatally (116/1,000) diagnosed. For all four anomalies, the median length of hospital stay was significantly greater in children with a prenatal diagnosis than those postnatally diagnosed. Children with prenatally diagnosed spina bifida (79% vs 60%; p = 0.002) were more likely to have surgery in the first week of life, with an indication that this also occurred in children with CDH (79% vs 69%; p = 0.06).

**Conclusions:**

Our findings do not show improved outcomes for prenatally diagnosed infants. For conditions where prenatal diagnoses were associated with greater mortality and morbidity, the findings might be attributed to increased detection of more severe anomalies. The increased mortality and morbidity in those diagnosed prenatally may be related to the lower mean gestational age (GA) at birth, leading to insufficient surfactant for respiratory effort. This is especially important for these four groups of children as they have to undergo anaesthesia and surgery shortly after birth. Appropriate prenatal counselling about the time and mode of delivery is needed.

**Supplementary Information:**

The online version contains supplementary material available at 10.1007/s10995-024-03911-9.

## Significance

**What is already known on this subject?:**

Prenatal detection of congenital anomalies has become more common with improvements in technology and increased accuracy of detection. Previous findings on the effect of timing of diagnoses on newborn outcomes are limited and conflicting.

**What this study adds?:**

Our findings do not show better outcomes for prenatally diagnosed infants with spina bifida, transposition of great arteries, congenital diaphragmatic hernia or gastroschisis. More severe anomalies may be more likely to be prenatally detected. Appropriate prenatal counselling about the time and mode of delivery is needed.

**Supplementary Information:**

The online version contains supplementary material available at 10.1007/s10995-024-03911-9.

## Introduction

Prenatal detection of congenital anomalies has become more common in recent decades, with improvements in ultrasound technology and the associated increased accuracy of detection (Peyvandi et al., 2016). The increasing prenatal detection rate of congenital anomalies may also reflect the growing number of scans during pregnancy (Mesas Burgos et al., 2016). Differences in detection rates may be attributable to the availability and quality of ultrasound equipment, the experience of the examiner, the type of congenital anomaly, and the gestational age of the fetus at examination (Skari et al., 1998). Detection of congenital anomalies before birth allows better planning of prenatal and postnatal care (Gamba and Midrio 2014; Grivell et al., 2014). In recent years, it has also allowed prenatal fetal therapy to be performed in rare cases (Sadlecki and Walentowicz-Sadlecka, 2023). Often, however, prenatal diagnosis seems not to result in better outcomes, but instead predicts poorer outcomes, possibly due to the more severe cases being more likely to be detected prenatally (Peyvandi et al., 2016; Lazar et al., 2011). There is also a need to balance the benefits of a prenatal diagnosis with lower gestational age following an induced preterm birth. For example, elective delivery of fetuses with complex congenital heart defects at early term (37–38 weeks) by induction or planned caesarean section results in lower gestational age and lower birth weight, both of which affect morbidity and mortality (Costello et al., 2014; Ho et al., 2021; Goldstein et al., 2022). Since previous findings on the effect of timing of diagnoses are limited and conflicting, more research on the subject is needed.

This EUROlinkCAT study (Establishing a linked European Cohort of Children with Congenital Anomalies) compared mortality and hospital care of prenatally and postnatally diagnosed infants with four specific isolated congenital anomalies: spina bifida, transposition of great arteries (TGA), congenital diaphragmatic hernia (CDH) and gastroschisis. These four congenital anomalies are well-defined major anomalies that are generally diagnosed prenatally as part of prenatal screening programmes in most countries, and the neonates need treatment immediately or shortly after birth.

## Materials and Methods

### Inclusion Criteria

Ten EUROCAT (European Surveillance of Congenital Anomalies network) registers are included in this study: Finland, the Northern Netherlands, Emilia-Romagna and Tuscany (Italy), Zagreb (Croatia), and Valencian Region (Spain), and East Midlands & South Yorkshire, Thames Valley, Wales, and Wessex (United Kingdom). Nine of the ten registers linked to vital statistics, while one registry (Zagreb) manually linked cases to mortality and hospital discharge records.

### Data File from the EUROCAT Registry

Cases for the study were all live born infants from 23 weeks of gestational age, between 1995 (or the year of inception of the registration, if later) and 2015 with isolated spina bifida, TGA, CDH and gastroschisis as defined in the EUROCAT Guide 1.4 (https://eu-rd-platform.jrc.ec.europa.eu/eurocat/data-collection/guidelines-for-data-registration_en#inline-nav-2). All children with associated anomalies and/or with genetic anomalies were excluded. Isolated anomalies were defined as structural anomalies within a single organ system only or as part of a sequence, as defined using the EUROCAT algorithm (Garne et al., 2011). For example, a child with TGA and ventricular septal defect (VSD) would be considered as having an isolated TGA, since TGA and VSD are part of the same organ system.

### Health Care Databases

Each EUROCAT registry linked their own data to mortality data and to the hospital episode data collected in the local or national hospital databases using either a personal identification number (ID) or linked through common variables (date of birth, gestational age, birth weight, sex, maternal age/date of birth), as described elsewhere (Loane et al., 2021). Consecutive hospital treatment periods were combined to one episode. The data on each episode included information on admission and discharge dates, diagnoses and surgical procedure(s), as registered in the primary source. The number of hospital days was calculated by the difference between discharge and admission date (Urhoj et al., 2022). To be confident that children with no recorded hospitalisations during the study period were indeed not hospitalised, rather than that their hospitalisations were not reported due to missed linkages, all children were also linked to other databases (i.e. national statistics, vital statistics, hospital databases outside the study period, and outpatient records) and only children with successful linkage to these other databases were included in the analysis (Table 1). All hospital obstetric stays were excluded from the study. In addition, infants who died ≥ 1 day after birth and were not identified in hospital databases were also excluded: 1.4% of all children not linked to hospital or other databases (Loane et al., 2023). For each child, data were extracted for the first 10 years of life or up to December 31st, 2015 for all registers, except children in Northern Netherlands born since 2013, who were followed to December 31st, 2017.

**Table 1:** The proportion of children with congenital anomalies that were linked to their national/ vital statistics or hospital databases and the number of live births according to anomaly

	% Children linked to national/vital statistics or hospital databases	Number of live births with isolated anomalies			
		Spina bifida	TGA	CDH	Gastroschisis
Croatia, Zagreb (2008–2014)	46.8	4	5	0	1
Finland (1997–2014)	99.4	84	273	76	155
Italy, Emilia Romagna (2008–2014)	92.8	220	66	46	16
Italy, Tuscany (2005–2014)	87.6	9	68	38	16
The Netherlands, North (LMR)^a^ (1995–2010)	95.4	45^b^	95^b^	40^b^	15^b^
The Netherlands, North (LBZ)^a^ (2013–2014)	94.9	0^b^	5^b^	0^b^	5^b^
Spain, Valencian Region (2010–2014)	98.9	14	27	17	12
UK, Wales (1998–2014)	98.9	60	131	76	235
UK, England, East Midlands & South Yorkshire (2003–2012)	89.4	91	214	131	291
UK, England, Thames Valley (2005–2013)	87.6	23	77	40	65
UK, England, Wessex (2004–2014)	85.2	24	96	46	124
Total number of live births		376	1057	510	935

^a^ EUROCAT Northern Netherlands linked to two healthcare databases: LMR (Landelijke Medische Registratie) and LBZ (Landelijke Basisregistratie Ziekenhuiszorg)

^b^ All results rounded to the nearest five

### The Linked Data

The linked data files were stored securely, either within the local register or within the organization doing the linkage. The registers received centrally written STATA syntax scripts to standardise the data to a common data model (CDM) and to create pre-specified tables and analyse the data (Appendix 1). Analytic results and aggregate tables were uploaded to a secure web portal for download to the Central Results Repository (CRR) at the University of Ulster. No individual case data were transmitted to the CRR.

The registers and databases of Northern Netherlands and Wales have governance restrictions regarding extracting and reporting data involving small numbers of cases. In the Northern Netherlands, all exported results must be rounded to the nearest five. In some tables, concerning small numbers of cases, the rounding of the data from the Northern Netherlands greatly influenced the overall estimates. In Tables 2 and 6, the cases from the Northern Netherlands were excluded.

### Core Outcomes from the Health Care Databases

The main outcomes for this study were: (1) survival to one year of age (divided into early neonatal mortality [deaths within the first 6 days of life] and infant mortality [deaths within the first year of life]); (2) reported surgical procedures within the first year of life; (3) the number of days spent in hospital under the age of five years. Both mortality and surgical procedures were analysed during the first year of life rather than up until age 5, as relatively few deaths and surgical procedures occur in children with these isolated anomalies after their first year of life compared with the great numbers occurring during the first year of life. Therefore, the power to detect any significant differences in later years would be very low (Glinianaia et al., 2022). Similarly, hospitalisations between the age 5 and 10 were not used in our study.

The numbers of days in hospital for each admission was calculated as the difference between the date of admission and date of discharge and were summed over all admissions for the child during the specified age ranges of under 1 year and 1–4 years. The median lengths of stay per year were calculated, adjusted for censoring on December 31st, 2015. In addition, the proportion of children with at least one single stay in hospital longer than 10 days or longer than 21 days was calculated separately.

Surgical procedures were coded according to the coding systems used in the national health systems. Procedures performed in the first year of life were identified based on the local coding system: Italy and Spain used ICD-9-CM (9th version of International Classification of Diseases—Clinical Modification) for the study period, Wales and England used OPCS-4 (The OPCS Classification of Interventions and Procedures) and Finland used a national adaptation of NCSP (NOMESCO Classification of Surgical Procedures). Since data on surgical procedures were not available for the Northern Netherlands, the cases from this register were excluded from the analyses. All codes were reviewed by paediatricians, as these coding systems also includes diagnostic and therapeutic procedures. The occurrence of surgical procedures during infancy was analysed by separating surgical procedures performed in the first week of life (0–6 days) from those performed later (7–364 days).

### Statistical Methods

For each congenital anomaly, the numbers of deaths were aggregated across the registers, according to whether the children were diagnosed prenatally or postnatally. The death rates were compared using Fisher’s exact tests. No adjustment was made for register due to the extremely small numbers involved. The mean gestational age at birth was also compared, for each congenital anomaly, between children who were prenatally diagnosed and those postnatally diagnosed by fitting meta-regression models on the mean and standard deviation of the gestational ages, from each register, with a variable indicating whether the mean was from the prenatally or postnatally diagnosed children. The mean follow-up duration (calculated to fifth birthday) was 4.2 person-years for EUROCAT children and 4.3 person-years for reference children.

For each register, the proportion of children admitted to hospital within each age group (i.e. under 1 year and 1–4 years) was calculated using Kaplan–Meier survival analysis to allow for the censoring of children occurring on December 31st 2015, death or emigration from the study region or country. 1) Cox’s proportional hazards models were used to calculate the hazard ratios (HRs) for ‘ever admitted to hospital’ and 2) negative binomial regression models were used to calculate the incidence rate ratios (IRRs) for number of days in hospital under the age of one year and 1–4 years, comparing prenatally diagnosed with postnatally diagnosed.

The confidence intervals for the proportions of children admitted to hospital in the Kaplan–Meier survival analysis estimates were calculated by STATA (version 16) using the ln(-ln(S(t))) transformation. To obtain pooled estimates of the proportion of children admitted across registers, the random effects inverse-variance meta-analyses were performed using the ln(-ln(S(t))) transformation and the I^2^ statistic calculated (using I^2^ = 100x(*Q − df)/Q*, where *Q* is the Cochran's homogeneity test statistic and *df* is the degrees of freedom). Since, in several registers, all children with a specific anomaly were admitted to hospital before the age of one year, the Kaplan–Meier survival analysis in STATA did not provide estimates of the confidence intervals. Here, the lower confidence interval was calculated using the exact binomial estimates with the number of live births as the denominator; the proportion of children admitted was estimated to be 99.9% and the upper 95% confidence interval was calculated assuming symmetry on the ln(-ln(S(t)) scale. The same analyses were performed for the proportions of children who had a single hospital stay of 10 days or more, or 21 days or more. Pooled estimates of the HRs and IRRs comparing prenatally diagnosed with postnatally diagnosed cases, within each register, were obtained using random effects meta-analysis. Additionally, separate analyses were undertaken by birth cohort: 1995–2004, 2005–2009 and 2010–2014. All meta-analyses were performed using the “metan” package in Stata, version 16.

## Results

Linkage success was high at over 85% for all registers, except in Zagreb, where the linkage was manually performed and only 47% of the children were linked (Table 1). The full dataset included 376 children with spina bifida, 1057 children with TGA, 510 children with CDH, and 935 children with gastroschisis. Information on when the anomaly was diagnosed was missing for 7 (1.9%) with spina bifida, 55 (5.2%) with TGA, 16 (3.1%) with CDH and 14 (1.5%) with gastroschisis, and these cases were excluded from the analyses. The percentage of live births with a known prenatal diagnosis was highest for children with gastroschisis (96%) and lowest for children with TGA (38%). Approximately half of the children with spina bifida (55%) or with CDH (56%) had been prenatally diagnosed (Table 2).

**Table 2:** Mean gestational age, early neonatal (0–6 days) and infant mortality (< 1 year including 0–6 days), number and rate per 1000 live births with 95% confidence intervals (excluding data from the Northern Netherlands)

Congenital anomaly	Prenatally diagnosed	No. of Live births (%)	Mean gestational age and 95% CI (weeks)	Early neonatal period (0–6 days)			Whole Infant Period (0–364 days)		
				Number of deaths	Mortality per 1000 live births (95% CI)	Comparison prenatal vs postnatal	Number of deaths	Mortality per 1000 live births	Comparison prenatal vs postnatal
Spina bifida	Yes	174 (55)	38.3 (37.8–38.7)	2	11 (1–41)	P = 0.50	3	17 (2–50)	P = 0.25
	No	145 (45)	39.1 (38.7–39.6)	0	0 (0–25)		0	0 (0–25)	
Transposition of great arteries	Yes	342 (38)	38.6 (38.3–39.0)	7	20 (8–42)	P = 0.40	39	114 (92–153)	P = 0.096
	No	555 (62)	39.2 (38.9–39.6)	17	31 (18–49)		44	79 (58–105)	
Congenital diaphragmatic hernia	Yes	256 (56)	37.5 (36.9–38.0)	71	277 (223–337)	P < 0.001	92	359 (301–421)	P < 0.001
	No	198 (44)	38.7 (38.0–39.3)	15	76 (43–122)		23	116 (75–169)	
Gastroschisis	Yes	861 (96)	35.9 (35.4–36.4)	4	5 (1–12)	P = 1.0	22	26 (16–38)	P = 1.0
	No	40 (4)	36.7 (35.9–37.5)	0	0 (0–88)		1	25 (0–132)	

For all four anomalies, children whose anomaly was detected prenatally had a lower mean gestational age than those detected at birth or later (Table 2). The gestational age was lower for live born children with gastroschisis (35.9 weeks for prenatally diagnosed vs. 36.7 weeks for those postnatally diagnosed, p = 0.089), and higher for children with CDH (37.5 vs. 38.7 weeks, p = 0.011), spina bifida (38.3 vs. 39.1 weeks, p = 0.011), and TGA (38.6 weeks vs. 39.2 weeks, P = 0.026).

Table 2 shows that both early neonatal mortality and infant mortality were highest for children with CDH and lowest for children with spina bifida and gastroschisis. Mortality was significantly higher in children with CDH who were diagnosed prenatally compared with those diagnosed later; early neonatal mortality for children with CDH was 277 vs. 76 per 1000 (p < 0.001) and infant mortality was 359 vs. 116 per 1000 (p < 0.001). These differences remained when gestational age at birth was included in the analysis. Neonates with CDH, born before 37 weeks of gestation, had higher mortality if diagnosed prenatally than when diagnosed postnatally (465/1000 vs 190/1000, p < 0.001) as did neonates with CDH born at term (271/1000 vs. 83/1000, p < 0.001). For all other anomalies there were no significant differences in mortality among preterm newborns in those diagnosed prenatally compared with those diagnosed at birth or later.

**Fig. 1:**
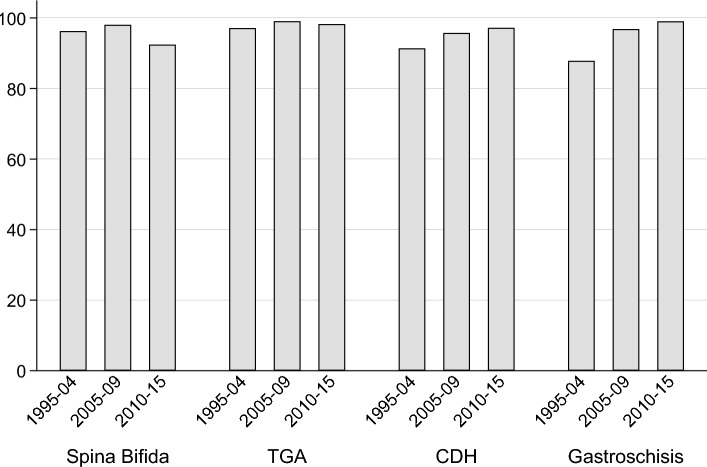
The proportion of children hospitalised under the age of one year, according to congenital anomaly and birth cohort

All children born with one of these four specific anomalies require surgical treatment in hospital in their first year of life to survive. During the study period, the proportion of children hospitalised in the first year of life was over 93% for all four selected anomalies, and these proportions (except for children with spina bifida) increased to over 98% by 2010–2014 (Fig. 1).

Table 3 shows that the median length of stay during the first year of life and for age 1–4 years was significantly greater in children with a prenatal diagnosis than those diagnosed later. The relative risks of spending an additional day in hospital reflected this, being significantly higher for children with prenatal diagnoses. For example, children with CDH under one year of age with prenatal diagnoses were almost 2.5 times more likely to spend an additional day in hospital compared with those diagnosed later (IRR = 2.44; 95% CI:1.81–3.28). Children aged 1–4 years spent much less time in hospital (the median length of stay per year was under two days for all four anomalies compared with over 17 days in the first year of life). Therefore, the absolute differences in number of days spent in hospital were smaller in those with prenatal diagnoses compared with those diagnosed later, but the relative risks were greater. Excluding gastroschisis, the proportions of children being admitted to hospital in years 1–4 is greater for children with prenatal diagnoses than those diagnosed later, and a statistically significant difference was observed for children with TGA (Table 4).

**Table 3:** Median number of days in hospital before one year of age and between 1–4 years according to prenatal diagnosis, with incidence rate ratio of days in hospital and percentage with a stay of more than 21 days

		Children hospitalised before the age of 1 year			Children hospitalised between the ages of 1–4 years		
Anomaly	Prenatally diagnosed	Median days per year (Lower Quartile—Upper Quartile)	IRR for hospitalisation (95% CI)	Percentage in hospital with a stay of more than 21 days (95% CI)	Median days per year (Lower Quartile—Upper Quartile)	IRR for hospitalisation (95% CI)	Percentage in hospital with a stay of more than 21 days (95% CI)
Spina bifida	Yes	17.5 (17.0–22.0)	1.83 (1.32–2.54)	36.3 (29.1–43.5)	1.7 (0.6–3.9)	1.32 (1.20–1.44)	8.3 (4.0–12.6)
	No	16.7 (10.5–30.5)	1	31.1 (23.2–39.0)	1.4 (0.5–3.3)	1	2.5 (0.0–5.4)
Transposition of great arteries	Yes	27.0 (25.75–30.0)	1.44 (1.11–1.86)	53.9 (45.6–59.3)	1.4 (0.4–3.4)	2.38 (2.03–2.73)	9.3 (5.9–12.6)
	No	22.0 (22.0–25.0)	1	45.3 (41.1–49.6)	0.8 (0.3–2.1)	1	4.2 (2.4–6.1)
Congenital diaphragmatic hernia	Yes	28.0 (19.0–28.0)	2.44 (1.81–3.28)	50.7 (44.2–57.1)	0.9 (0.3–3.9)	1.42 (1.31–1.53)	3.8 (0.5–7.1)
	No	21.0 (13.7–23.0)	1	46.2 (38.9–53.4)	0.6 (0.2–14)	1	0.7 (0.0–1.9)
Gastroschisis	Yes	34.2 (34.0–39.0)	1.15 (0.76–1.73)	82.1 (79.5–84.7)	1.0 (0.4–3.2)	3.11 (3.00–3.23)	3.7 (2.3–5.0)
	No	28.2 (26.0–36.2)	1	77.1 (63.2–91.1)	0.2 (0.1–0.3)	1	0

*CI* confidence interval

*IRR* incidence rate ratio estimated from Negative Binomial Regression of length of stay in hospital with exposure being days in study before upper age limit or censored on 31/12/2015, whichever occurred first

**Table 4:** Hospitalisations before the age of one year and between 1–4 years: numbers of cases and proportions according to prenatal diagnoses with hazard ratios (HR) for hospitalisation in children prenatally diagnosed compared with those later diagnosed

Anomaly	Prenatally diagnosed	Children hospitalised before the age of 1			Children hospitalised between the ages of 1–4		
		N	%	HR for hospitalisation (95% CI)	N	**%**	HR for hospitalisation (95% CI)
Spina bifida	Yes	191	98.1 (93.3–99.5)	1.13 (0.97–1.32)	151	93.6 (89.9–97.4)	1.05 (0.98–1.13)
	No	157	92.8 (81.9–97.2)	1.0	116	88.9 (83.5–94.3)	1.0
Transposition of great arteries	Yes	347	97.8 (92.2–99.4)	1.15 (0.99–1.32)	199	76.8 (71.6–81.9)	1.26 (1.14–1.39)
	No	616	98.6 (94.3–99.6)	1.0	291	61.0 (56.6–65.4)	1.0
Congenital diaphragmatic hernia	Yes	239	96.7 (89.9–98.9)	1.05 (0.94–1.18)	91	66.7 (58.8–74.6)	1.05 (0.89–1.25)
	No	207	92.9 (86.0–96.5)	1.0	100	63.3 (55.8–70.8)	1.0
Gastroschisis	Yes	830	97.6 (94.6–99.0)	0.97 (0.94–1.00)	399	54.6 (51.0–58.2)	0.83 (0.64–1.09)
	No	40	95.9 (75.2–99.4)	1.0	20	65.6 (48.7–82.5)	1.0

*HR* Cox Proportional Hazard Ratio—ever admitted to hospital

The proportion of children having at least one hospital stay of 21 days in their first year of life was higher for children diagnosed prenatally in all four anomaly groups, with the differences being statistically significant only for children with TGA (Table 3). The same pattern was observed for children aged 1–4 years, but the differences were statistically insignificant. A similar pattern is also seen when analysing hospital stays of 10 days or more among children aged 1–4 years, but here the difference for children with TGA was statistically significant (Appendix Table 6).

**Fig. 2:**
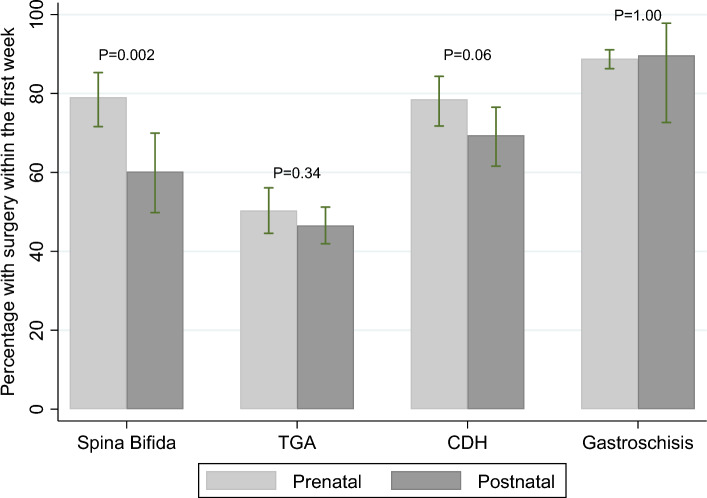
Percentage with a surgical procedure during the first week of life (with 95% confidence intervals) in children with prenatal and postnatal diagnoses

For children with spina bifida with a surgical procedure in the first year, those diagnosed prenatally more often had their first surgical procedure during their first week of life (79%) than children with postnatal diagnosis (60%) (p = 0.002) (Fig. 2). For children with CDH, the difference did not reach statistical significance (79% vs. 69% for children with prenatal diagnoses vs later diagnoses, p = 0.061). There was no association between timing of diagnoses and timing of first surgical procedure for children with TGA and gastroschisis, with surgery in the first week of life for 50% and 47% with prenatal and postnatal diagnosis of TGA, and 89% and 90% of cases with prenatal and postnatal diagnosis of gastroschisis (Table 5).

**Table 5:** Numbers and proportions of children undergoing first surgical procedure before the age of one week and one year (excluding data from the Northern Netherlands)

Anomaly	Prenatal diagnosis	No. with surgery		Percentage having surgery in first week (%)	Comparison Prenatal diagnosis
		Within 1st week	Within 1st year		
Spina bifida	Yes	117	148	79	P = 0.002
	No	59	98	60	
Transposition of the great arteries	Yes	153	304	50	P = 0.34
	No	215	462	47	
Congenital diaphragmatic hernia	Yes	139	177	79	P = 0.06
	No	109	157	69	
Gastroschisis	Yes	629	708	89	P = 1.00
	No	26	29	90	

## Discussion

In this study the prenatal detection rate among live births was highest for gastroschisis (96%), lowest for TGA (38%) and similar for spina bifida (55%) and CDH (56%). Children with a prenatal diagnosis were born approximately one week earlier and had longer stays in hospital during their first five years of life than children diagnosed after birth. Children with CDH had a higher neonatal and infant mortality, when prenatally diagnosed. One explanation may be that prenatally detected children may have more severe anomalies that are more readily visible on an ultrasound scan (Peyvandi et al., 2016, Costello et al., 2014). Children with CDH and spina bifida diagnosed prenatally were more likely to have their first surgical procedure within the first week of life, which may reflect more rapid and efficient planning of postnatal care subsequent to the perinatal diagnoses.

For spina bifida, our results and postulation that prenatally detected children may have more severe subtypes of this congenital anomaly are consistent with the guidelines for the Society of Obstetricians and Gynaecologists of Canada (Douglas Wilson et al., 2021). These recommend that as the ability to detect the severity of the cases through prenatal diagnosis has increased, specialist units should plan the mode of delivery, and plan and undertake immediate and subsequent postnatal care. Prenatal diagnosis can be used to plan the mode of delivery, but for those with spina bifida, the choice between elective caesarean section and vaginal delivery after spontaneous labour did not seem to affect motor function or ambulation status (Lewis et al., 2004). A randomised trial reported in 2011 concluded that myelomeningocele repair in utero by hysterotomy reduced the need for a shunt placement within the first year and also improved motor outcomes at 30 months compared with the standard postnatal repair (Adzick et al., 2011). However, the data from our study covers the years 1995 to 2014 and very few such prenatal surgical procedures were likely to have occurred in the children in our data.

For CDH there have been several studies reporting either no association or a negative association between a prenatal diagnosis and subsequent mortality and morbidity (Mesas Burgos et al., 2016; Skari et al., 1998; Colvin et al., 2005; Barriere et al., 2018; Long et al., 2019) and concluding that observed association were due to the prenatally diagnosed cases having more severe condition. A recent RCT showed that prenatal treatment of severe CDH (Deprest et al., 2021a), but not moderate CDH (Deprest et al., 2021b) improved newborn outcomes. A study of 30 cases with CDH (Bétrémieux et al., 2004) apparently contradicts these findings by showing that prenatally diagnosed isolated CDH treated with immediate planned intensive care had a higher survival than those diagnosed at birth or later or those not receiving the full specifically adapted postnatal procedure. However, this study evaluated the effectiveness of the immediate planned care as well as the effectiveness of prenatal diagnosis (Bétrémieux et al., 2004). The big difference in mortality found in this study between prenatal and postnatal diagnosis may be explained by inclusion of infants with CDH diagnosed weeks or months after birth. All these children are expected to survive their surgery and their mortality rate was 25.3%. Mortality for prenatally diagnosed CDH cases was 35.9% which is consistent with the literature, while it was 11.9% for postnatally diagnosed cases, which is lower than expected. Infants who died ≥ 1 day after birth not identified in hospital databases were excluded from our study to avoid bias due to “missing” linkage. It is possible that some infants diagnosed with CDH postnatally may have died before being transferred to a tertiary hospital for treatment, and these deaths (most of which occurred in the first 28 days of life) were excluded from the study (Loane et al., 2023). The European Reference Network ERNICA has proposed a protocol that recommends using standardized ultrasound assessments of fetuses with isolated CDH to predict the neonatal outcome (Russo et al., 2018).

Studies have reported either lower mortality and morbidity in children with prenatally diagnosed TGA compared with postnatally diagnosed or no association of prenatal diagnoses with outcome (Bonnet et al., 1999; Cloete et al., 2019; Kunde et al., 2021; Nagata et al., 2020; van Velzen et al., 2015; Domínguez-Manzano et al., 2017). Postnatal acidosis and need for intubation have been reported to be more common among postnatally diagnosed cases, but many longer-term outcome measures, for example the length of post-operative Intensive Care Unit (ICU) stay, have not differed between the groups. It has been suggested that prenatal diagnosis of TGA is associated with better neurocognitive outcomes, especially executive function (Calderon et al., 2012; Peake et al., 2015). We found that prenatally diagnosed cases were more likely to spend more time in hospital up to the age of 5 years, but, in agreement with other studies, there was no clear difference in surgical procedures within the first week of life.

Prenatal diagnosis of gastroschisis does not seem to be associated with a poorer outcome when compared with postnatal diagnosis (Sipes et al., 1990). However, there are indications that certain prenatal ultrasound findings (intra-abdominal bowel dilatation, polyhydramnios and gastric dilatation) can be used to identify neonates with a higher risk of postnatal complications (D'Antonio et al., 2015). In our study, over 90% of cases were detected prenatally, resulting in a small sample of cases detected postnatally, which limited the power of our study to detect any associations with a prenatal diagnosis.

### Strengths and Limitations

A strength of this study was that data were standardised to a CDM by each participating register. Further, all registers are population-based and are members of EUROCAT and therefore the quality of the coding of the congenital anomalies is high and all births with the specified anomalies in areas covered by the registers are included. Many published studies are based on children presenting to tertiary care centres, which may yield biased samples by excluding the less severely affected children. It may be a limitation of this study that the diagnosis of an isolated TGA may be a proxy for a more complex heart anomaly, since there may be differences in how TGAs are coded across the participating registers.

The registration of hospitalisations and surgical procedures in a hospital database, and the access to surgical care may differ in time and place. Moreover, children with severe and complex congenital anomalies may have been referred for specialised surgical or medical treatment outside the registry area before or after birth, which may underestimate the morbidity. We expected that all children with the selected congenital anomalies would be treated in hospital unless they died before receiving a unique ID and/or being registered in the hospital database. For all four selected anomalies the proportion of children hospitalised in the first year of life increased from 93% or more to over 98% (except for children with spina bifida) during the study period. Thus, the proportion of neonates with the selected four congenital anomalies with a hospital stay or a surgical procedure recorded in our study is likely to be a slight underestimate of the true number requiring such care in early life.

A weakness of our study was that we could not receive individual patient data from the registers as this was a linkage study using secondary data sources and there were governance restrictions on the onward sharing of data. Therefore, all analyses had to be pre-specified, and it was not possible to investigate specific results in greater detail. In particular, it was not possible to confirm the assumption that the children with prenatal diagnoses had more severe anomalies than those detected at birth or later as there was no access to the individual medical records. Further, we were not able to adjust our results for potential confounders due to differences in the availability and comparability of relevant background characteristics. Differences in morbidity and mortality may be impacted by changes and advances in prenatal care and in care of infants with congenital anomalies over the study period. While screening policies and practices vary over time and between counties, only three registries contributed data for the earlier years in our study (1995–1999). We were unable to assess the impact of advances in health care in our study.

The data from EUROlinkCAT has been shown to be reliable and misclassification bias is unlikely to have occurred, However, linkage was not 100% and an earlier study demonstrated that live births resulting in deaths in the first week of life were less likely to be linked. However, the data in this study were restricted to those registries with very high linkage success (apart from Zagreb) and therefore bias is unlikely to have arisen due to missed linkages. Zagreb contributed only ten cases and therefore their data are unlikely to bias the overall results. Finally, even though our data were based on ten registers, the sample sizes were small, especially for some outcomes such as death. Neither did we have information on the quality of information on prenatal diagnoses in these registers, but the data are provided to EUROCAT and their quality is assumed to be good (see: https://eu-rd-platform.jrc.ec.europa.eu/eurocat/data-collection/data-quality_en).

This study showed that for CDH and spina bifida, children with anomalies diagnosed prenatally appeared to have lower gestational ages, higher rates of mortality and morbidity and undergo surgery earlier than those diagnosed at birth or later. A prenatal diagnosis appeared to make less difference to children with TGA or gastroschisis, although they spent longer in hospital. From a clinical perspective, it seems plausible that outcomes would be improved if the diagnosis is known before birth: e.g., the pregnant woman can be referred to a tertiary hospital for childbirth at the optimal gestational age and specialist clinicians can care for the severely ill neonate immediately after birth. For those diagnoses, where prenatal diagnoses correlated with higher mortality and morbidity, the findings were probably due to the more severe anomalies being more likely to be detected. An important finding in our study is that gestational age at birth is lower for children diagnosed prenatally. Lower gestational age may contribute to the increased morbidity shortly after birth due to immaturity of the respiratory system, and because transition at birth may take longer. This is especially important for these four groups of children as they have to undergo anaesthesia and surgery shortly after birth. Earlier gestational age at birth in the prenatally diagnosed infants may be related to the presence of a more severe type of anomaly. When assessing the potential benefits of any early intervention, treatment protocols for these infants should be informed by the possible harms of labour induction and early elective caesarean section (such as reduced breastfeeding rates, for example Jordan et al., 2009), compared with watchful waiting for spontaneous childbirth. For all four congenital anomalies, appropriate prenatal counselling about the time and mode of delivery is needed.

### Electronic supplementary material

Below is the link to the electronic supplementary material.

Supplementary file1 (DOCX 92 KB)

## Data Availability

Due to data protection issues data are not available. The data have been collected for this specific study with specific permissions. Each site can be contacted to get more detailed information, how similar data can be received for research purposes.

## Appendix

See Table 6.

**Table 6:** Number and proportion of children with a stay of more than 10 days in hospital, by age at admission

Anomaly	Prenatal diagnoses	No. children	Proportion in hospital < 1 year (95% CI)	p-value	No. children	Proportion in hospital 1 – 4 years (95% CI)	p-value
Spina bifida	Yes	124	63.2 (51.0–73.1)	0.04	27	17.2 (11.3–23.1)	0.10
	No	90	51.5 (35.8–65.2)		12	10.2 (4.7–15.6)	
Transposition of great arteries	Yes	306	85.6 (75.9–91.6)	0.60	61	21.7 (16.9–26.5)	0.001
	No	515	83.2 (77.6–87.6)		60	12.7 (9.7–15.7)	
Congenital diaphragmatic hernia	Yes	167	66.5 (58.7–73.1)	0.45	10	7.6 (3.1–12.2)	0.18
	No	157	69.3 (59.7–77.0)		6	4.0 (0.9–7.0)	
Gastroschisis	Yes	786	92.7 (86.9–96.0)	0.72	36	4.7 (3.2–6.2)	0.23
	No	37	87.5 (56.6–96.9)		0	–	
